# Taurine suppresses the spread of cell death in electrically coupled RPE cells

**Published:** 2008-10-29

**Authors:** Chandani Udawatte, Haohua Qian, Nancy J. Mangini, Brian G. Kennedy, Harris Ripps

**Affiliations:** 1 Departments of Ophthalmology and Visual Sciences, University of Illinois College of Medicine, Chicago, IL; 2 Anatomy and Cell Biology, University of Illinois College of Medicine, Chicago, IL; 3 Physiology and Biophysics, University of Illinois College of Medicine, Chicago, IL; 4 Biological Sciences, University of Illinois College of Medicine, Chicago, IL; 5 Indiana University School of Medicine-Northwest, Gary, IN

## Abstract

**Purpose:**

To determine whether taurine exerts a protective effect on retinal pigment epithelium (RPE) cells exposed to a cytotoxic agent, cytochrome C (cyC), shown previously to induce apoptosis and produce cell death in electrically coupled neighboring cells.

**Methods:**

Monolayer cultures of confluent human RPE (ARPE-19) cells, which express gap-junctional proteins, were incubated in culture medium with or without taurine. After scrape loading cyC into the cells, we assayed these cells for caspase 3 activity and terminal deoxynucleotidyl transferase dUTP nick end labeling (TUNEL) staining to determine the spread of apoptosis.

**Results:**

We found that cyC, too large a molecule to traverse gap junctional channels, produced apoptosis in cells injured by the scrape as well as those distant from the site of the scrape, presumably by the intercellular transmission of a toxic agent through the gap junctions that couple these cells. Incubation in taurine, or the gap-junction blocker, octanol, before application of cyC, reduced significantly the fraction of cells undergoing apoptosis. Voltage clamp recordings from electrically coupled *Xenopus* oocytes transfected with Cx43 showed that junctional communication was unaffected by taurine.

**Conclusions:**

Our results indicate that taurine can serve to suppress cell death in RPE cells independent of any effect on gap junctions. We have considered various avenues by which taurine can exert its protective effect, but the precise mechanism involved under these experimental conditions has yet to be identified.

## Introduction

The present study was prompted by a growing number of reports advocating the use of taurine and related compounds as therapeutic agents for a wide range of disorders that induce apoptosis in tissues throughout the body [1-3]. For example, it has been shown that taurine serves as a free radical scavenger and an antagonist to oxidative stress in protecting heart, lung, and liver cells from cell death [4-7], and it has proven useful as an anticonvulsant in reducing epileptic seizures [8]. In addition, there is good evidence that taurine, one of the major constituents of the mammalian central nervous system, is essential for normal retinal development [9,10]. The concentration of taurine in the distal layers, including photoreceptors and retinal pigment epithelium (RPE), of the vertebrate retina is estimated to be 60–80 mM [11-13]. Although taurine’s precise function has often been conjectural, numerous studies have shown that a taurine-deficient diet, or the inhibition of taurine transport, causes photoreceptor loss and RPE abnormalities in a variety of animal species including primates [9,14-17].

Interestingly, despite the high oxygen consumption required to meet the energy demand of cells of the distal retina, more proximal retinal layers exhibit a greater susceptibility to metabolic or hypoxic/ischemic insult [18]. Indeed, it appears likely that photoreceptors and RPE cells are rendered resistant to metabolic insufficiencies by an endogenous agent that serves either to prevent apoptosis or to suppress the spread of cell death across the layers of cells that constitute the RPE and the photoreceptors, each of which is linked to its neighbors by gap junctions [19-21]. In the present study we sought to determine whether taurine can exert a protective effect on RPE cells using the human RPE (ARPE-19) cell line as a model system.

To address these issues we modified a scrape-loading technique used earlier to study the spread of apoptosis through gap-junctional channels [22,23]. The method involves the introduction of cytochrome C (cyC) to trigger downstream caspase activity in a limited population of cultured cells, i.e., those opened to the extracellular milieu by the scrape, and to then assay by immunocytochemistry and terminal deoxynucleotidyl transferase dUTP nick end labeling (TUNEL) staining the spread of apoptosis to neighboring cells through the gap junctions with which they are coupled. The procedure enabled us to examine the effects of taurine on the induction and spread of apoptosis in an ARPE-19 immortalized cell line derived from human RPE [24]. Because of the possibility that taurine inhibits gap junction intercellular communication (GJIC), we determined whether taurine affects GJIC between *Xenopus* oocytes electrically coupled through heterologous expression of the RPE gap junctional protein, Cx43.

## Methods

### Reagents

The sources from which we obtained primary and secondary antibodies for immunocytochemistry are indicated in the text; serum-free media (Neurobasal) was from Gibco (Invitrogen, Carlsbad, CA); all other chemicals were analytical grade or better, and purchased from Sigma-Aldrich, St. Louis, MO.

### Cell line

Cultures of ARPE-19 cells, provided by Dr. Beatrice Yue (Department of Ophthalmology, University of Illinois College of Medicine, Chicago, IL) were seeded at a density of roughly 1 × 10^4^ cells/cm^2^ in polystyrene dishes with a glass bottom (Becton Dickinson, Franklin Lakes, NJ). The medium was changed every 3–4 days, and the cells were grown to confluency in a normal growth medium, consisting of Dulbecco’s modified Eagle’s medium (DMEM) supplemented with 10% fetal bovine serum and 1% penicillin and 1% streptomycin (1% PS). Cells were kept in a 37 °C incubator under a humidified 5% CO_2_ : 95% air atmosphere, and were dissociated twice weekly by mild treatment with 0.25% trypsin and 0.02% ethylenediaminetetraacetic acid (EDTA) and subcultured in the growth medium.

### Western blot analysis

ARPE-19 cells were grown to confluence on 25 cm^2^ Primaria tissue culture flasks (Becton Dickinson). Cells were removed by scraping and solubilized with SDS sample buffer, which contained 2% SDS, 10% glycerol, 200 mM HEPES at pH 6.8, 1 mM EDTA, 0.1% bromphenol blue, and 5% 2-mercaptoethanol. The buffer was supplemented with a protease inhibitor cocktail (Roche Diagnostics, Indianapolis, IN). Samples were resolved by SDS–PAGE on 6% Laemmli gels (20 µg total protein per lane), and transferred to PVDF membranes. Membranes were blocked with Super Block (Pierce Biotechnology, Inc., Rockford, IL) and exposed to rabbit anticonnexin 43 antibody (Zymed Laboratories Inc., San Francisco, CA; catalog number 71–0700) at 1:500 dilution in high sodium TBS buffer that contained 500 mM NaCl and 25 mM Tris buffer, pH 7.5. Cross-reacting bands were visualized using antirabbit alkaline phosphatase secondary antibody (Vector Laboratories, Burlingame, CA) and chemiluminescent detection using Immunobilon Western AP Substrate (Millipore, Bedford, MA). Signals were captured with the Image Station 440 CF New England Nuclear (NEN, Boston, MA) and analyzed with Kodak 1D Image Analysis Software.

### Connexin expression

**Figure 1:**
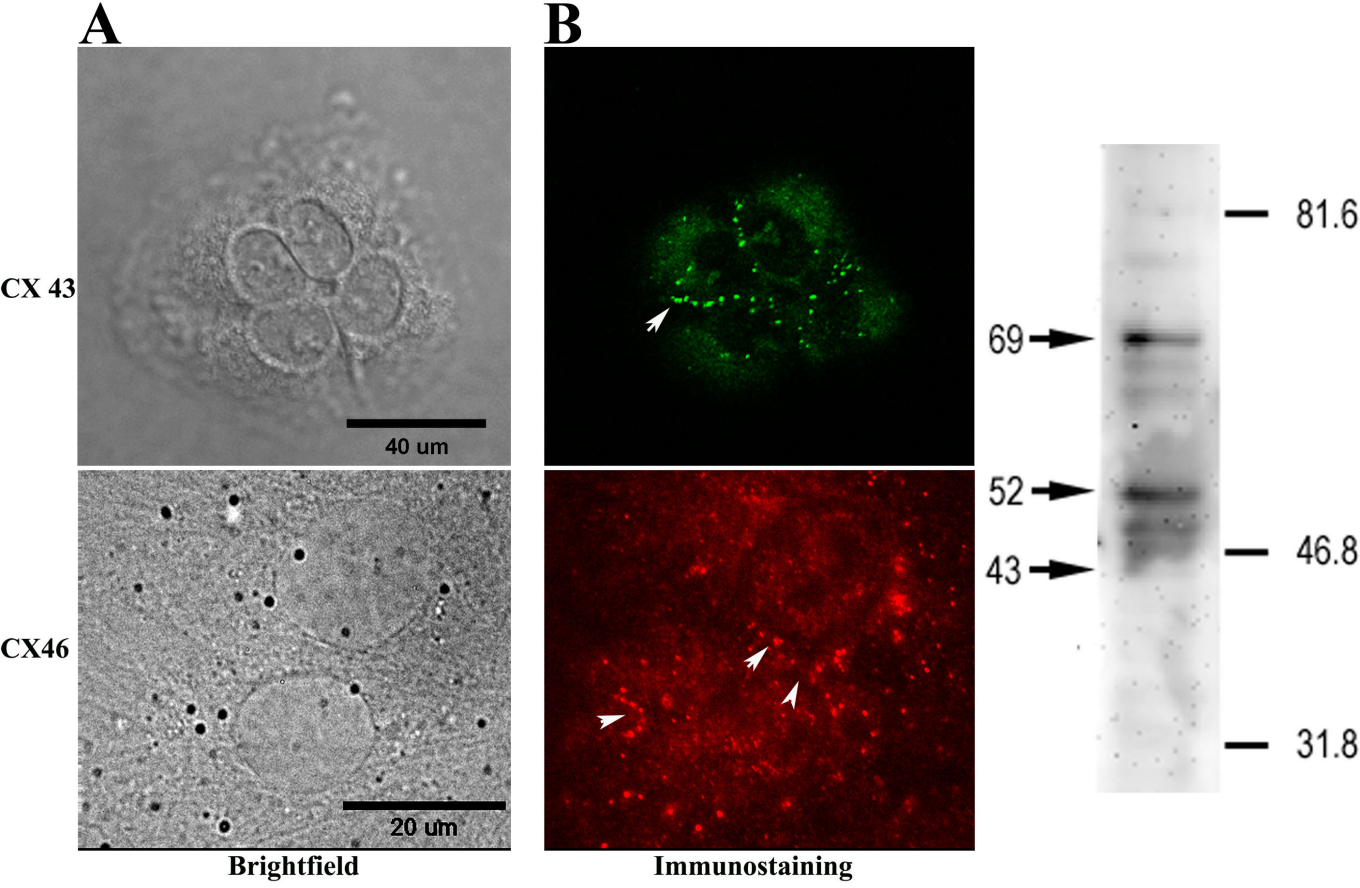
Connexin expression in ARPE-19 cells. **A:** Immunocytochemistry: an antibody to connexin 43 was revealed with Alexa 488-tagged secondary antibody, and the antibody to connexin 46 was visualized by fluorescent microscopy with a cy3-tagged secondary antibody. Punctate labeling characteristic of connexin plaques was seen both in the region of cell apposition (arrows) and at the cell surface. In the left panel, scale bar in the Differential interference contrast (DIC; upper) image represents 40 μm, while scale bar in the phase (lower) image represents 20 μm. **B:** Western blotting: Aliquot of whole cell ARPE-19 lysate (20 µg total protein) were resolved by SDS–PAGE on a 6% Laemmli gel and transferred to PVDF membrane. Anti-Cx43 antibody labeled several bands corresponding to phosphorylated species of Cx43 (major bands identified by arrows at 69 and 52) and a minor band corresponding to nonphosphorylated Cx43 (arrow at 43). Numbers and lines to the right indicate the positions of molecular weight markers.

The cellular localization of Cx43 and Cx46, gap junctional proteins expressed by ARPE-19 cells [20,25,26] was examined by immunocytochemistry (Figure 1). Connexin expression and GJIC are prerequisites for the intercellular spread of cell death by toxic agents generated during apoptosis in our experimental protocol [22,23]. Cells were plated on sterile 35 mm plastic dishes, grown to near confluence, washed with PBS (17 mM KH_2_PO_4_, 5 mM Na_2_HPO_4_, 150 mM NaCl), and fixed either with 4% paraformaldehyde in PBS, or in buffered methanol, which was composed of 90% methanol, 10 mM HEPES, 0.1 mM MgCl_2_, and 0.1 mM EGTA, chilled to −20 °C. Similar findings were obtained with both fixatives and will not be considered further in the Results section. The cells were then permeabilized in 0.1% Triton-X-100 for 10 min at room temperature then blocked for 2 h at 37 °C in a solution containing either 3% fetal calf serum or 10% goat serum in PBS. They were incubated overnight at 4 °C in a polyclonal rabbit anticonnexin 43 antibody against a synthetic peptide corresponding to a segment of the third cytoplasmic domain of rat connexin43 (Zymed Laboratories Inc.). Antibody binding was visualized by fluorescent microscopy using goat antirabbit Alexa 488 secondary antibody (Molecular Probes, Inc., Eugene, OR) as well as with a sheep antirabbit FITC secondary antibody (Jackson ImmunoResearch Laboratories, West Grove, PA). Cells were similarly examined for the expression of connexin 46, by incubating with a polyclonal rabbit anticonnexin 46 antibody (a gift from Dr. Nalin Kumar, Department of Ophthalmology, University of Illinois College of Medicine, Chicago, IL), and a donkey antirabbit cy3 secondary antibody (Jackson ImmunoResearch Laboratories). Differential interference contrast (DIC), phase contrast, and fluorescence images were collected using either a Zeiss Axiovert 100 M microscope (Zeiss, Oberkochen, Germany) or an Olympus Fluoview 300 confocal microscope.

### Intercellular communication

**Figure 2:**
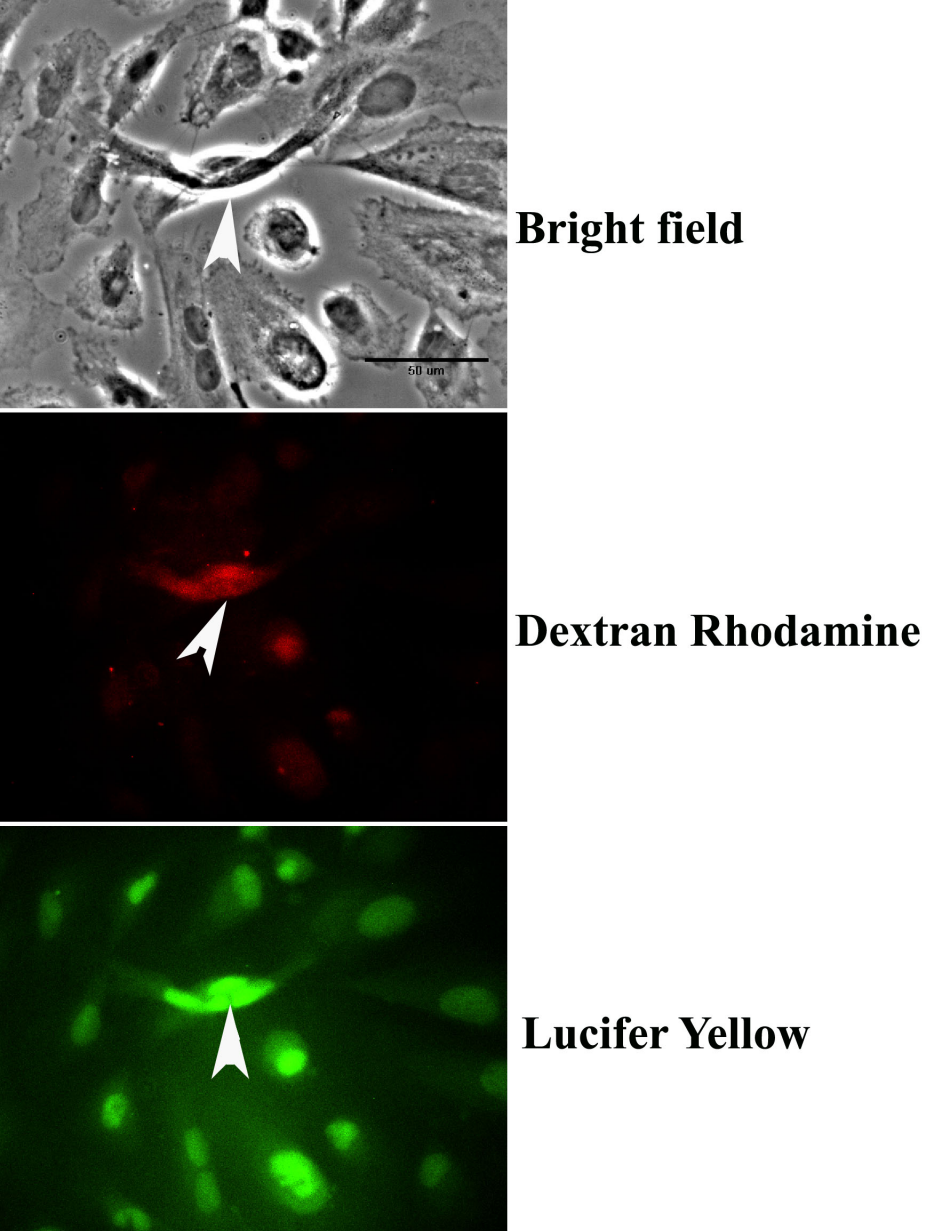
Dye spread in electrically coupled cells. Gap junctions mediate the spread of Lucifer yellow (LY), indicating that junctional permeability and the system of intercellular communication are intact. Arrowheads in all panels show the location of the small injury (scrape) produced by the tip of the scalpel blade. Fluorescent images of cells incorporating both dextran rhodamine (DR) and LY show that whereas the rhodamine complex did not spread from the site of the scrape (middle panel), LY spread through the gap junctions that coupled the cells (lower panel). Scale bar (upper panel) equals 50 μm.

To determine whether the RPE cells expressing connexins Cx43 and Cx46 formed communicating junctions with their neighbors, we gently cut a region of the monolayer with the point of a scalpel blade. The cells were then bathed for 5 min in 1 ml PBS solutions supplemented with 100 μl of a 0.05% solution of Lucifer yellow (LY; Sigma) and 100 μl of 5 mg/ml solution of dextran rhodamine B, 10000 MW, neutral (DR; Molecular Probes Inc.). Thereafter, the LY-DR solution was exchanged for the normal growth medium, and the cells were incubated at 37 °C for 1 h. LY is able to traverse the aqueous pores of gap junctions, which typically allow the passage of ions and small molecules ≤1 kDa [27-29], whereas the DR complex can only enter the cells that were injured by the scrape. The cells were fixed briefly (approximately 15 min) with 4% paraformaldehyde, washed once with PBS, mounted with Vectashield (Vector Laboratories,), and examined by fluorescent microscopy (Figure 2).

### Scrape loading cytochrome C and apoptotic assays

**Figure 3:**
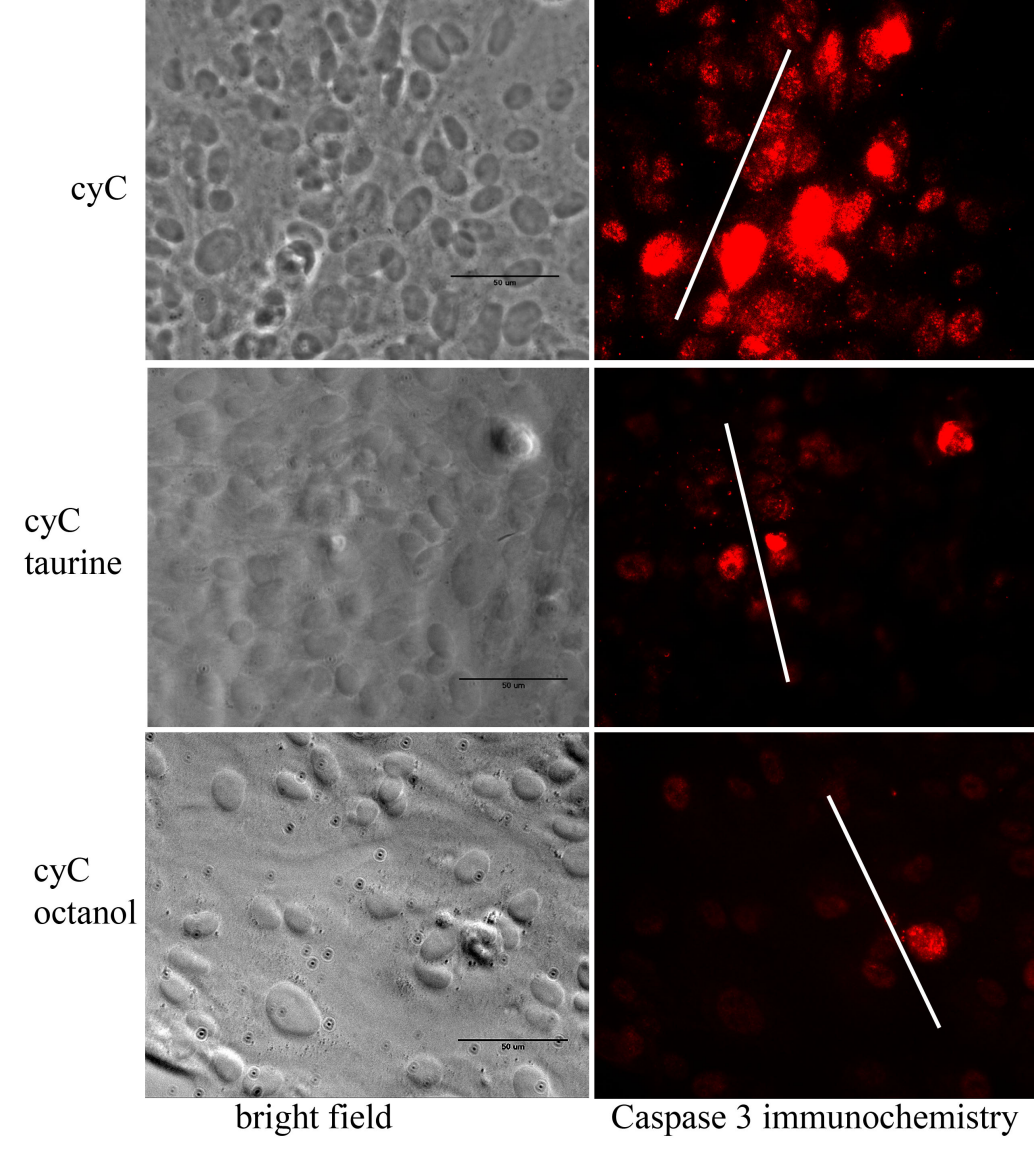
Cell death revealed by caspase 3 activity. In the absence of taurine or the gap-junctional channel blocker octanol, an antibody to active caspase 3 shows that cell death induced by incubation with cytochome C (cyC) was widespread, extending to cells beyond the scrape (upper panels). In contrast, cell death in the presence of taurine (middle panels) or octanol (lower panels) was limited primarily to cells along the lines of the scrape. White lines indicate the position and approximate extent of the scrape. Scale bars represent 50 μm.

**Figure 4:**
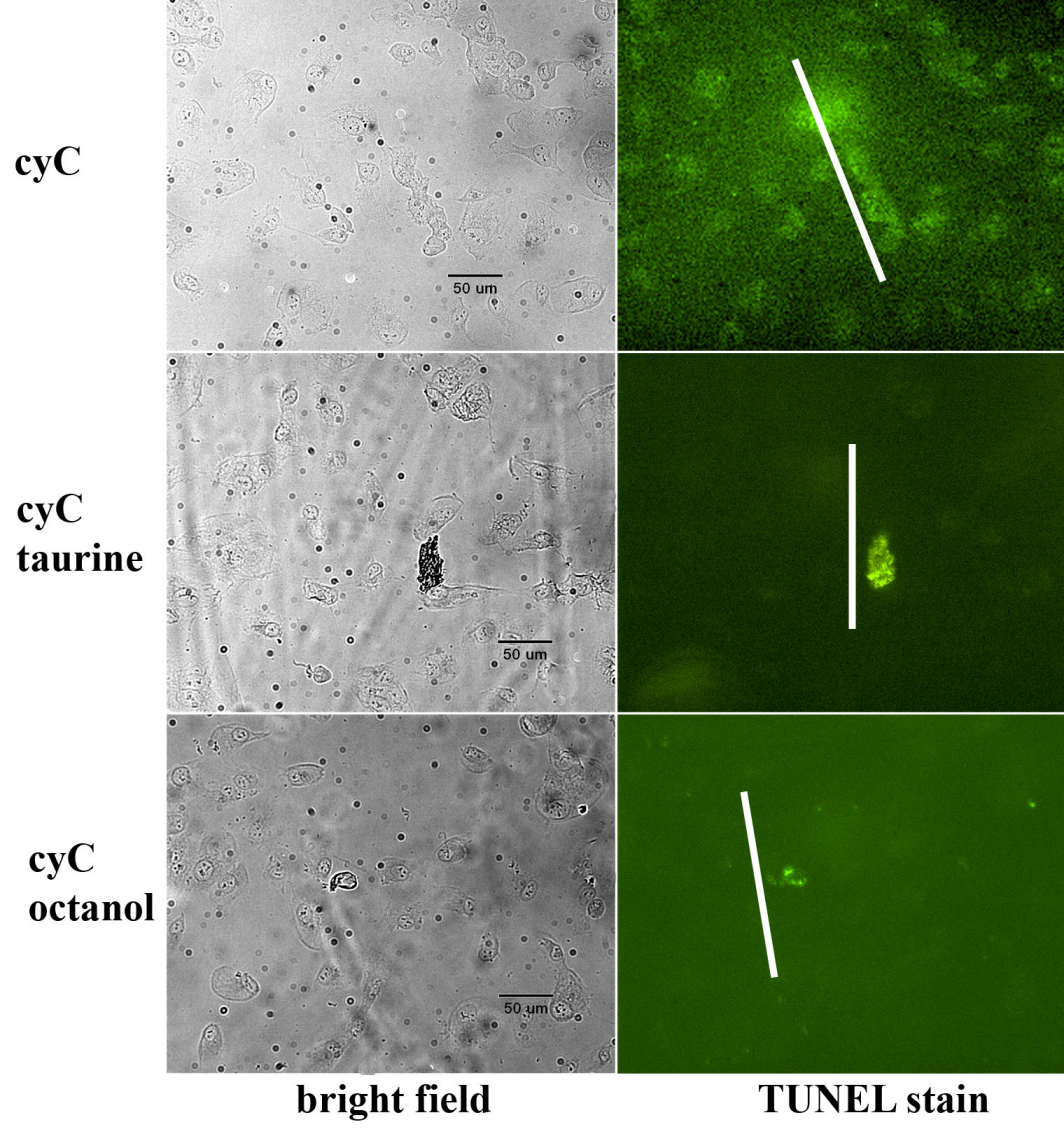
Apoptosis visualized by TUNEL. TUNEL staining of ARPE-19 cells incubated in the presence of cytochrome C (cyC) with (middle panels) or without (upper panels) exposure to taurine also showed that significantly fewer cells were undergoing cyC-induced apoptosis in the presence of taurine. Octanol had a similar effect to taurine in blocking the spread of cell death induced by cyC (lower panels). Scale bars represent 50 μm.

Cells grown to confluency in 35-mm plastic dishes were incubated overnight in DMEM supplemented with 10% FBS and 1% PS, either with (test) or without (negative control) the addition of 20 mM taurine. Additionally, cells were incubated with 10 mM octanol (Sigma), a substance known to block intercellular gap junctional communication. We did not attempt to determine whether the ARPE-19 cells have an endogenous supply of taurine, but there is evidence that despite numerous passages this cell line contains a taurine transporter to promote the entry of extracellular taurine [30,31]. After removing the growth medium, we immersed the cells in a PBS solution containing 100 μl of 1 mM cyC, and a gentle touch with the blade of a mechanical tissue chopper produced the “scrape” into which cyC entered the damaged cells. In this connection, it is important to note that cyC (12.3 kDa) is neither membrane permeable nor can it traverse the pores of gap-junctional channels. The cells were incubated in the cyC solution at room temperature for 5 min after which growth medium containing 20 mM taurine was added to the cyC solution bathing the “test” cells, which were then incubated in the mixture for 2 h at 37 °C. Cells exposed to octanol were treated in the same manner as taurine-incubated cells, and they were incubated at 37 °C for 2 h in growth medium containing octanol after induction of apoptosis with cyC. After repeated rinses in PBS to remove the cyC, the cells were fixed in 4% paraformaldehyde overnight at 4 °C and prepared for histochemical analysis. In examining the figures depicting caspase 3 activation and TUNEL staining (Figure 3 and Figure 4), it is important to recognize that most of the cells incorporating cyC immediately adjacent to the scrape had died and do not appear in the figures, having been washed away in the rinses used to remove the cyC.

### Cell death assays

#### Caspase 3 activity and TUNEL staining

Cysteine-dependent aspartate-directed proteases (caspases) play a prominent role in apoptosis [32,33], and caspase 3 activity provides a reliable reporter of cells undergoing apoptosis; DNA degradation revealed by TUNEL staining is also considered to be a major indicator of apoptosis. The procedures for these and related assays of apoptosis have been described previously [22]. Briefly, immunocytochemistry for the presence of caspase 3 activity was performed using a rabbit polyclonal antibody against cleaved caspase 3 (Cell Signaling Technology, Beverly, MA). The caspase 3 antibody was diluted 1:100; the secondary antibody was donkey antirabbit cy3 (Jackson ImmunoResearch Laboratories).

In the TUNEL assay, partially degraded DNA is labeled with fluorescein-dUTP by using terminal deoxynucleotidyltransferase (TdT) to introduce the nucleotide preferentially into 3′ strand breaks [34]. TUNEL label, a nucleotide mix containing fluorescein-dUTP and dNTP was obtained from Roche Applied Science (Indianapolis, IN). After induction of apoptosis by cyC, the cells were treated according to the manufacturer’s instructions for TUNEL staining. The cells were washed three times with PBS and fixed with 4% paraformaldehyde in PBS for 1 h at room temperature. They were then rinsed with PBS, incubated for 2 min with an ice-cold permeabilization solution containing 0.1% Triton X-100 in 0.1% sodium citrate, and rinsed again with PBS. Next, 5 μl of TdT and 45 μl of TUNEL label solution were mixed well and added to the cells, which were allowed to incubate at 37 °C for 1 h in a closed humidified chamber. After two rinses in PBS, the cells were mounted in Vectashield and viewed under the fluorescent microscope.

#### Image acquisition and processing

**Figure 5:**
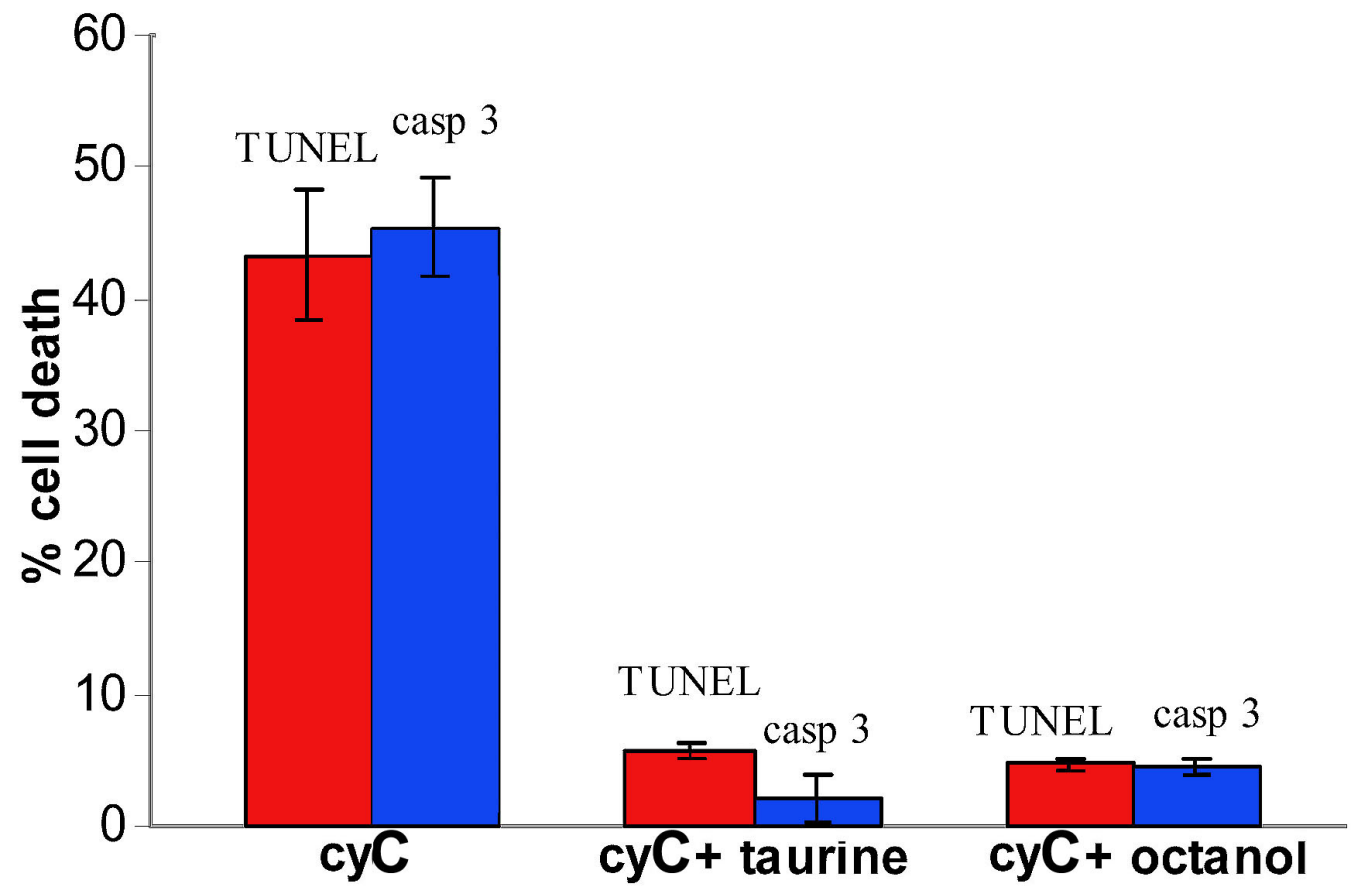
Quantification of cell death. Percent cell death was determined with activated caspase 3 immunocytochemistry and TUNEL assay as described in Methods. The percentage cell death was reduced by approximately 80% in the presence of taurine or the gap junctional blocker octanol, (p<0.001, comparing taurine- or octanol-treated group means to respective cyC-only control means; ANOVA with Student-Newman-Keuls post hoc testing). The reduction in percentage cell death in the presence of taurine was statistically indistinguishable from that in the presence of octanol (p>0.05). Error bars depict SEM; n=4 for caspase activity and n=3 for the TUNEL assay. “casp 3” refers to activated caspase 3 immunocytochemistry and “cyC” refers to cyctochrome C.

Cells examined for dye transfer and immunocytochemistry were visualized with a Zeiss Axiovert 100 M microscope (Zeiss) through either a plan-Neofluar 20X/0.5 or an Achroplan 40X/0.6 phase objective, and photographed with a SensiCam CCD camera (resolution 1280x1024; Cooke Corp., Auburn Hills, MI). Image processing was controlled by MetaMorph software (Universal Imaging Corp., Westchester, PA). For each of the assays indicated, we determined the number of cells staining for a specific marker within a 9.6 × 10^5^ µm^2^ field that included the site of the scrape. Computing the ratio of stained cells to the entire cell population within the field gave the percentage values indicated by the histograms shown in Figure 5. Statistical analysis was performed using Student’s *t*-test; differences at p<0.05 were considered significant.

### Connexin expression in *Xenopus* oocytes

#### Oocyte preparation and electrophysiological recording

Plasmids containing the coding sequence of rat Cx43 were generously provided by Drs. David Spray (Albert Einstein College of Medicine, New York, NY) and Thomas White (State University of New York, Stony Brook, NY). The construct was linearized with a restriction endonuclease (Xba1), and capped mRNA was transcribed in vitro with T7 RNA polymerase using the mMessage mMachine (Ambion Inc., Austin, TX) according to the manufacturer’s instructions. Ovarian lobes were removed under surgical anesthesia (0.1% MS222; tricaine, ethyl 4-aminobenzoate) from gravid Xenopus laevis females purchased from Xenopus I (Dexter, MI). The animals were housed in climate-controlled, light-cycled rooms at the UIC Biological Resources Laboratory, and fed standard frog chow. All experimental procedures adhered to the guidelines for the care and use of laboratory animals formulated by the Association for Research in Ophthalmology and Visual Sciences, and were approved by the Animal Care Committee of the University of Illinois at Chicago College of Medicine. The lobes were incubated with constant agitation for 2 h in a calcium-free modified Barth’s (MB) solution containing 2.5 mg/ml collagenase. Defolliculated stage V-VI oocytes were selected and repeatedly rinsed in MB solution that contained 88 mM NaCl, 1 mM KCl (1), 2.4 mM NaHCO_3_,15 mM N-2-hydroxyethylpiperazine-N’-2-ethanesulfonic acid (HEPES), 0.33 mM Ca (NO_3_)_2_, 0.41 mM CaCl_2_, and 0.82 mM MgSO_4_; 10 mg/liter gentamycin (Gibco/BRL) was added, and the solution titrated with NaOH to pH 7.4. After overnight incubation in MB at 15 °C, oocytes were injected with 46 nl of an aqueous solution containing 10 ng or 20 ng/cell Cx43 cRNA using a Nanoject Injector (Drummond Scientific Co., Broomall, PA); 10 ng of an antisense oligonucleotide was added to the solution to suppress the activity of the endogenous connexin (Cx38) of *Xenopus* oocytes [35]. Cells receiving the antisense oligo alone served as controls.

Oocytes were tested 48-72 h after cRNA injection for the effects of taurine on gap junctional conductance. The cells were manually stripped of the vitelline envelope in hypertonic medium [36], mounted on Teflon platforms within a Lucite chamber (volume=0.7 ml), and paired at their vegetal poles for 48 h before electrophysiological analysis using a dual amplifier and dual electrode voltage clamp technique [37]. Solutions were superfused at a rate of 12 ml/minute using a multiport gravity feed system (MP6 manifold; Warner Instrument Corp., Hamden, CT), and fluid was withdrawn through a suction pipette; solution exchange within the chamber was complete in <8 s. Taurine was added to the MB solution in concentrations of 20–40 mM without substitution; no dose-dependent differences were observed. Current and voltage electrodes (R=1–2 MΩ) were filled with a solution containing 3 M KCl, 10 mM EGTA, and 10 mM HEPES, pH 7.4, and connected to the input stages of two GeneClamp 500B amplifiers (Axon Instruments, Foster City, CA).

**Figure 6:**
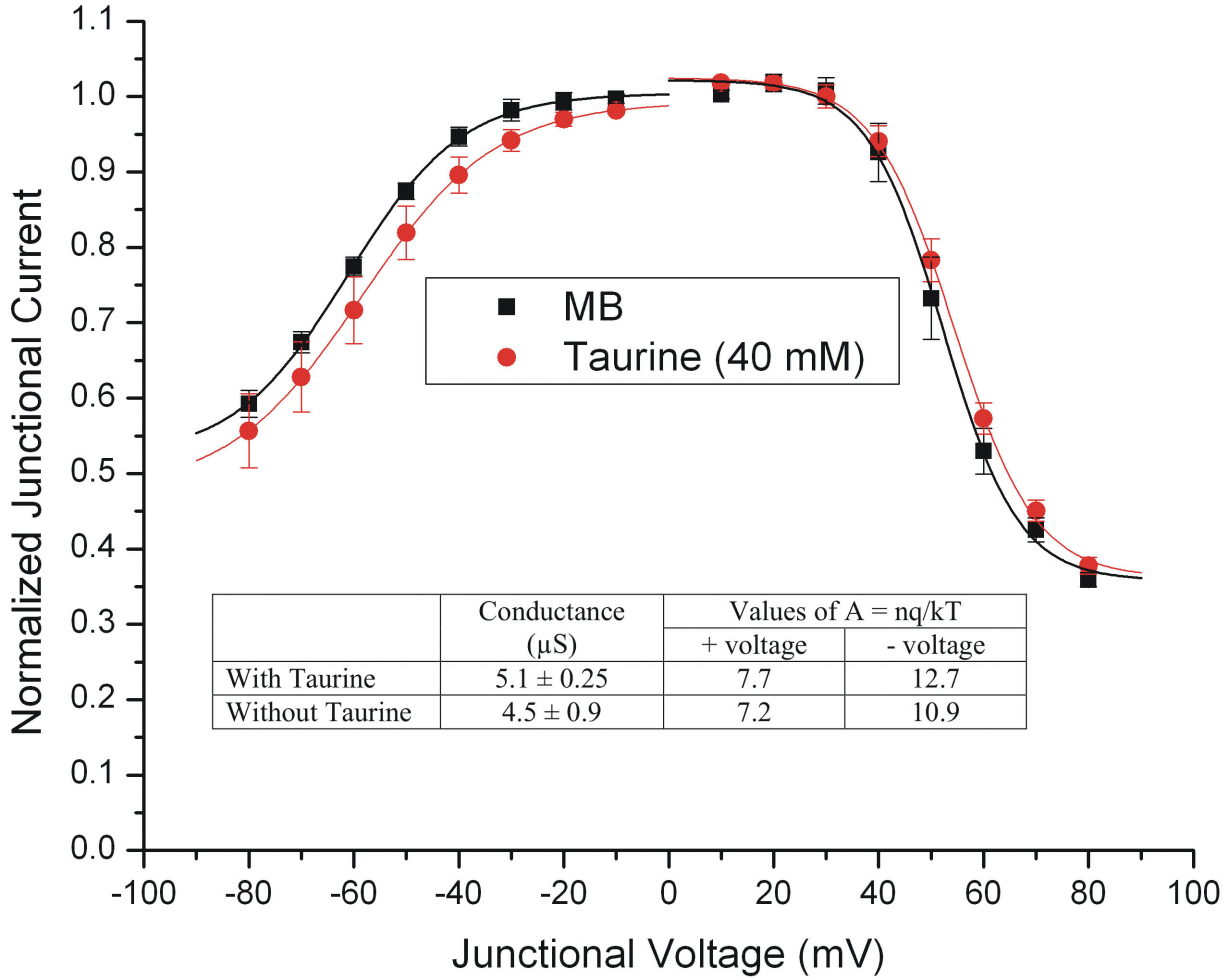
Taurine effect on junctional conductance. Exposure to 40 mM taurine had no significant effect on the voltage sensitivity of the junctional currents recorded over the range of ±80 mV, although there was a slight current reduction at negative junctional voltages. The data (n=4 for each condition) were fit by the Boltzmann equation:  **G_jss_  =  { (G_jmax_ – G_jmin _) / ( 1 + exp [A ( V_j_ –V_o _) ] } + G_jmin_** where G_jmax_ (normalized to unity) is the maximum conductance, G_jmin_ is the residual conductance at large values of V_j_, and V_o_ is the V_j_ at which G_jss_ = (G_jmax_–G_jmin_)/2. The constant A (A=nq/kT) represents the voltage sensitivity in terms of gating charge as the equivalent number (n) of electron charges (q) moving through the membrane, k is the Boltzmann constant, and T is the absolute temperature. As shown in the inset, taurine had no significant effect on the junctional conductance between paired oocytes expressing this gap-junctional protein (p=0.5444 by Student’s *t*-test), and there were no significant differences in the values of A for taurine treated and control oocytes in either the positive or negative branches of the curves. These findings are a good indication that taurine does not affect Cx43 gap junctional channels.

Both cells of a pair were initially clamped at −40 mV (zero junctional potential, V_j_) and alternating pulses of ±10 mV were imposed to one cell. Current delivered to the cell clamped at −40 mV during the voltage pulse was equal in magnitude to the junctional current (I_j_) and was divided by the voltage to yield the conductance. The voltage-gating properties were determined as follows: V_j_ values of opposite polarity were generated by hyperpolarizing or depolarizing one cell in 10 mV steps (over a range of ±80 mV) while clamping the second cell at −40 mV. Currents were measured 8 s after the onset of the voltage pulse, at which time they typically reached steady-state (I_jss_), and the macroscopic conductance (G_jss_) was calculated by dividing I_jss_ by V_j_. G_jss_ was then normalized to the values determined at ±10 mV, and plotted against V_j_. Data describing the relationship of G_jss_ as a function of V_j_ were fit to a Boltzmann relation [38], additionally explained in the legend of Figure 6. The average conductance of oocyte pairs selected for analysis of voltage sensitivity was 4.8±0.6 μS (mean±SEM [standard error of the mean]). The experimental protocols were controlled by pClamp 8 software through a Digidata 1322A acquisition interface (Axon), and data analysis and graphical displays were performed with software programs in Clampfit 8 (Axon) and Origin 6.0 (MicroCal Inc., Northhampton, MA). Junctional conductance and current-voltage data show the means and variance (SEM) based on a minimum of 4 sets of recordings for each experimental condition.

## Results

Before examining the effects of taurine on the spread of cell death in cultured RPE cells, it was necessary to satisfy two requisite conditions: confirm the presence of connexins on RPE cells and show that the connexins form patent intercellular pathways in the two cell lines. With respect to connexin expression on RPE cells, earlier studies indicated the presence of Cx43 (the predominant form) and Cx46 on RPE cells using immunohistochemical [20], biochemical [25], and microarray analyses [26], and the functional properties of these intercellular junctions have been studied extensively [39-41]. The expression of Cx43 and Cx46 in the cells used in this study was examined with immunocytochemical and immunoblotting techniques. Typical results are shown in Figure 1. ARPE-19 cells exhibited the punctate labeling characteristic of connexin plaques on each of the RPE cells in the field of the microscope. Punctate staining along the margin between cells was especially clear with anti-Cx43, and Cx labeling at the cell surface was also evident. In this respect the labeling is similar to that seen on other non-neuronal cell types, e.g., astrocytes [42]. Immunoblot analysis further confirmed Cx43 expression on ARPE19 cells. As described previously, anti-Cx43 labeled multiple bands representing different phosphorylation states of the protein [25]. Prominent bands at approximately 69 kDa and 52 kDa are near the sizes reported for major upper and lower phosphorylated forms of Cx43; other minor bands were also detected. The arrow at 43 kDa identifies the position of a weak band at the size expected for the nonphosphorylated form of Cx43. Phosphorylation is implicated in the regulation of a variety of connexin processes, and the finding of multiple phosphorylated Cx43 species in ARPE19 cells suggests involvement of this connexin isoform in the formation of functional gap junction channels.

The gap junctions formed in apposed cells are permeable to LY, a tracer that provides an index of junctional permeability. As shown in the images in Figure 2, when a small incision is produced in the monolayer of ARPE-19 cells and then exposed to a solution containing LY and DR, cells remote from the site of injury were filled with LY. In contrast, the larger DR complex is confined to the site of the lesion. These results demonstrate that connexins are expressed in the cell line used for this study, and that the system of intercellular communication is intact.

Figure 3 illustrates the effects of taurine on the spread of cyC-induced apoptosis revealed by the antibody to cleaved caspase 3. These images show that before introducing taurine (upper panels), cell death spread to many cells remote from the site of injury (white lines in the images in the second column). However, in the presence of taurine (middle row of images), or by adding the gap-junctional blocker octanol (lower row of images), there is virtually no spread of cell death beyond the site of injury.

A similar result was obtained with the TUNEL assay for apoptosis. As shown in Figure 4, TUNEL staining was observed in cells remote from the location of the scrape in cells exposed to cyC only (upper panels). However in the presence of taurine (middle panels) or octanol (lower panels), TUNEL staining was observed almost exclusively in cells near the site of the scrape (white lines).

Cell death was assayed by determining the fraction of cells within each dish that displayed either caspase 3 activity or TUNEL labeling. Averaged data obtained in 3 (TUNEL) and 4 (caspase 3) separate experiments under each condition are illustrated in Figure 5. As shown in the bar graphs, taurine reduced cell death by more than 80% and was clearly as effective as the gap junction blocker octanol in this regard.

Although the effects of taurine and related aminosulfonates on gap junctions have been reported in numerous studies on a variety of different tissues, the results have not been consistent and often depend on the type of connexin expressed [43-46]. Thus it was necessary to consider the possibility that the beneficial effects of taurine observed under the present experimental conditions resulted from the blockage of gap-junctional conductance. However, we found that taurine did not affect significantly the junctional conductance between paired *Xenopus* oocytes expressing Cx43. As shown in the inset to Figure 6, conductance measurements on paired oocytes in MB (n=4) and in 40 mM taurine (n=4) showed no significant difference: the mean conductance in MB was 4.5±0.9 µS, and in the taurine solution it was 5.1±0.25 µS (p=0.5444 by unpaired t test; values are mean±SEM). In addition, the junctional currents (Figure 6), recorded over the range of ±80 mV, show that the voltage sensitivity was not significantly affected by this concentration of taurine. These findings are a good indication that any effects that taurine may have on cell death are not readily attributable to the blockage of gap junctional channels formed by Cx43. The evidence that taurine was without effect on gap-junctional communication mediated by Cx43, made it unnecessary to examine its effects on cell pairs expressing Cx46. Moreover, the study by Malfait et al. [25] suggests that Cx46 does not play a significant role in forming functional gap junctions in RPE. That aside, we recognize that the data were obtained from oocytes expressing Cx43, and may not accurately represent the situation in the cell lines.

## Discussion

Taurine (2-aminoethanesulfonic acid) is often referred to as a “conditionally- or semi-essential amino acid” because it is not used in protein synthesis. However, it is important to recall that taurine is ubiquitous in nature and is the most abundant amino acid in excitable cells and tissues throughout the body. It comprises a large percentage of the total free amino acids in the heart, kidney and plasma [47] and is vital for normal development [48-51]. It is present in high concentrations in skeletal muscle [52] and in liver, where it plays a significant role in conjugation of bile acids [53-56]. Many of the physiologic functions in which it has been implicated have yet to be clearly defined, but there is evidence that taurine interacts with various neurotransmitter systems [2], participates in membrane stabilization [1,57], and is effective in modulating numerous calcium-dependent processes [58,59]. In addition, there is new evidence indicating that taurine is able to inhibit apoptosis by preventing formation of the apoptosome [60], a key element in the deleterious action of mitochondrial-derived cytochrome C [61,62] leading to the caspase-mediated apoptotic cascade [32,63]. Clearly there are several avenues by which taurine can act to protect RPE cells from cell death, and they would explain in large part both the present experimental findings and the resistance of the outer retina to metabolic insult. Moreover, the loss of these properties may account for the multiple abnormalities in the visual, cardiovascular, and reproductive systems when taurine is depleted from the diet of animals deficient in cysteine sulfinic acid decarboxylase, an essential enzyme for the biosynthesis of taurine from cysteine or methionine [64]. Nevertheless, it remains to be seen whether the effects of taurine reported here result from one or more of the foregoing mechanism or by activating some other intracellular pathway that inhibits the cell death process.

Numerous studies have reported that cell death signals induced by cyC and other agents can be transmitted through the aqueous pores of gap junctions to adversely affect their neighbors [65-69], but that this so-called “bystander effect” [70] can be suppressed by blocking the gap junctional channels connecting the various cell types [22,68,71]. Thus there was the possibility that taurine exerts its beneficial effect by blocking the propagation of apoptosis through the intercellular channels that couple RPE cells, a possibility strengthened by reports that in some cell types gap junctional communication is inhibited by taurine [43,45]. However, as we have shown, the gap-junctional conductance of oocytes expressing Cx43 was not affected significantly by exposure to moderately high concentrations of taurine.

Although this study does not address directly a putative role for taurine in diabetic retinopathy, it is noteworthy that its effectiveness in suppressing organ lipid peroxidation [72] and in reducing insulin resistance [73] has led to the suggestion that taurine may be beneficial in preventing or ameliorating hyperglycemia-induced retinal defects [74-77]. In humans and in animal models, diabetes is consistently associated with a progressive increase in a range of abnormalities affecting the structural and functional properties of neurons and blood vessels of the *inner* retina [78-80]. These findings are in contrast to the seemingly milder effects of diabetes on the blood retinal barrier (BRB) and cells of the *outer* retina. Although there are reports of reduced amplitude electroretinogram potentials generated by the photoreceptors and RPE [81,82], as well as evidence of a small reduction in the thickness of the outer nuclear layer [83], the pathophysiological changes are neither as consistent nor as profound as those seen in the inner retina [79,84]. This is somewhat surprising considering the highly vascular capillary bed (choriocapillaris) that nourishes cells of the outer retina, the accumulation of glucose within the RPE in response to hyperglycemia [85], and the glucose transport properties of the photoreceptors [86] and RPE [87,88]. Clearly, the high concentrations of taurine in the RPE and photoreceptors may contribute to the relative sparing of the distal retina, which occurs despite the fact that hyperglycemia causes altered expression of the tight-junctional protein occludin [89], an increase in the lipid content of the RPE [90], and permeability defects in the RPE [91]. Nevertheless, the data on the beneficial effects of taurine supplementation in diabetes are still too sparse to be considered significant, and further biochemical and physiologic experimentation addressing this important issue is clearly warranted.

Lastly, it has not escaped our notice that the present results provide a possible mechanism for the protective effects of tauroursodeoxycholic acid (TUDCA) reported by Boatright et al. [3]. In their study, the authors provide convincing evidence that TUDCA, a taurine containing derivative of bile acid, protects photoreceptors from genetically mediated and photically induced cell death. This agent, which has been shown to prevent apoptosis in a broad spectrum of neurologic and systemic diseases [92,93], is deconjugated by the bacterial flora of the intestinal lumen to release taurine. Thus, it may not be inapposite to suggest that taurine could be the active substance underlying the therapeutic actions of TUDCA.

However, it is important to stress that in its unconjugated form, ursodeoxycholic acid (UDCA) has proven to be efficacious in the treatment of gallstones and cholestatic liver diseases [94]. When delivered subcutaneously, as done in the Boatright et al. study, UDCA may be equally effective in suppressing photoreceptor cell death.
